# Patient Questions and Physician Responses in a Chinese Health Q&A Website: Content Analysis

**DOI:** 10.2196/13071

**Published:** 2020-04-16

**Authors:** Ziying Hong, Zhaohua Deng, Richard Evans, Haiyan Wu

**Affiliations:** 1 School of Medicine and Health Management Tongji Medical College Huazhong University of Science and Technology Wuhan China; 2 School of Journalism and Information Communication Huazhong University of Science and Technology Wuhan China; 3 College of Engineering Design and Physical Sciences Brunel University London United Kingdom; 4 Undergraduate School of Medical Business Guangdong Pharmaceutical University Zhongshan China

**Keywords:** health information seeking, question classification

## Abstract

**Background:**

Since the turn of this century, the internet has become an invaluable resource for people seeking health information and answers to health-related queries. Health question and answer websites have grown in popularity in recent years as a means for patients to obtain health information from medical professionals. For patients suffering from chronic illnesses, it is vital that health care providers become better acquainted with patients’ information needs and learn how they express them in text format.

**Objective:**

The aims of this study were to: (1) explore whether patients can accurately and adequately express their information needs on health question and answer websites, (2) identify what types of problems are of most concern to those suffering from chronic illnesses, and (3) determine the relationship between question characteristics and the number of answers received.

**Methods:**

Questions were collected from a leading Chinese health question and answer website called “All questions will be answered” in January 2018. We focused on questions relating to diabetes and hepatitis, including those that were free and those that were financially rewarded. Content analysis was completed on a total of 7068 (diabetes) and 6685 (hepatitis) textual questions. Correlations between the characteristics of questions (number of words per question, value of reward) and the number of answers received were evaluated using linear regression analysis.

**Results:**

The majority of patients are able to accurately express their problem in text format, while some patients may require minor social support. The questions posted were related to three main topics: (1) prevention and examination, (2) diagnosis, and (3) treatment. Patients with diabetes were most concerned with the treatment received, whereas patients with hepatitis focused on the diagnosis results. The number of words per question and the value of the reward were negatively correlated with the number of answers. The number of words per question and the value of the reward were negatively correlated with the number of answers.

**Conclusions:**

This study provides valuable insights into the ability of patients suffering from chronic illnesses to make an understandable request on health question and answer websites. Health topics relating to diabetes and hepatitis were classified to address the health information needs of chronically ill patients. Furthermore, identification of the factors affecting the number of answers received per question can help users of these websites to better frame their questions to obtain more valuable answers.

## Introduction

### Background

Rapid developments in internet capabilities have led to public changes in attitudes toward information seeking. A Pew study conducted in 2012 reported that 59% of adults in the United States searched online for health information, and half of these searchers sought information on behalf of someone else [1]. Health care consumers seek information online relating to topics such as treatment, questions before or after visiting health care providers, or advice about diet and exercise habits [2,3]. Online health information plays a significant role in patients’ decision making as to how to manage a health issue or treat an illness [4,5]. During the past decade, health information has been made more readily available via the use of internet-enabled services, including social media sites [6], online communities, forums, and crowdsourcing mechanisms, to satisfy patients’ information needs [7].

With developments in information technologies, health-related question and answer (Q&A) websites have grown in popularity [8]. These sites are designed to allow people to ask and obtain responses to questions on a broad range of health-related topics [9], enabling patients to exchange information by posting questions and answers, thus providing a natural form of conversation in seeking information [10]. Obtaining information from health Q&A websites is beneficial for users owing to their relatively low cost (most services are free), quick turnaround time given the broad community participation, and easy build-up of social capital [11]. Three types of online Q&A services currently exist: (1) digital reference services, (2) expert services, and (3) social Q&A services [8].

Online health Q&A websites can be characterized as providing expert-based Q&A services according to the classification proposed by Shah et al [8]. On such websites, answers are provided by a group of health care experts rather than from an open community. The medical professionals are only allowed to answer questions once they have undergone a strict registration process, which requires certification and verification. Another distinguishing feature of expert-based Q&A services is that they often include a pricing system, and are thus generally referred to as a price-based knowledge market [12], such as JustAnswer [13] and PickAnswer [14].

Many popular health Q&A websites such as the “All questions will be answered” [15] service for general patients and the “Baby Tree” service [16] for expectant mothers or those raising a baby are available to both medical professionals and the general public. Online health expert Q&A websites focus on solving the health problems experienced by patients. People raise their health-related questions and receive professional responses such as information on the prevention and treatment of diseases [17] or guidance before and after meeting with a physician [18]. As a result of developments in internet technologies, health Q&A websites have become a convenient communication platform for both health professionals and patients seeking medical information [19]. By critically reviewing the questions posted on health Q&A websites, we can assist health care information providers to better understand the literacy skills and requirements of patients and their information needs.

Numerous models and theories exist to help understand the habits of those seeking health information. Griffin and Neuwirth [20] proposed a model for risk information seeking and processing. Afifi and Weiner [21] advanced the theory of motivated information. Longo [22] proposed a conceptual model to better understand the information-seeking behaviors of patients and consumers. These models delineate the reasons motivating patients to seek information and aim to predict the underlying process. However, these studies do not outline the necessary skills required by patients to complete the information-seeking process. These skills will affect the outcome of searches and subsequent health-related decision making, as health literacy influences health information seeking [23,24]. On health Q&A websites, consumers can express their health concerns in their natural language [25]. Although a patient’s ability to communicate their story and visualize the illness being experienced has been less acknowledged in research, this should be an important consideration for online health care environments [26]. In particular, there is limited knowledge as to how patients suffering from chronic illnesses are able to illustrate their situation and the problems they encounter in doing so. Further, explicit requests appear to be more successful than implicit requests [27]. In other words, stating the question explicitly, as a clear question, increases the number of responses received.

To address this issue, we explored the literacy of patients suffering from chronic illnesses in terms of their ability to describe their illness and express their question sufficiently on the online health Q&A website “All questions will be answered.”

### Research Question 1: Can Patients Suffering From Chronic Illnesses Describe Their Illness Accurately and Phrase Their Concerns Into a Question?

Increased understanding of patients’ preferences toward health care information, including how it is presented and delivered, is important for health care providers to determine the types of topics most sought by patients [28]. A review conducted by Ramsey et al [29] indicated that information related to a specific illness or disease is the most common type of information sought by consumers. Medical treatments or procedures [30], health care professionals [31], exercise and diets [32], and symptoms [33] are also frequently searched topics [29]. Patients with different diseases have varying information needs. For patients with cancer, for instance, the most frequently requested information is related to treatment [34]. Kuske et al [35] indicated that diet, complications, exercise, medications, and pharmacological interactions are the most frequently sought topics, which led us to our second research question.

### Research Question 2: What Topics Are Most Frequently Sought by Patients Suffering From Chronic Illnesses?

Many factors can potentially affect the quantity of responses received to a question. Teevan et al [36] identified that the punctuation used, number of sentences, and scope significantly affected the response rate. With reference to “question content” and “questioner characteristics,” Liu et al [37] identified 17 extrinsic factors that have a significant effect on a question’s response rate, including the posting style and time period. To accelerate the time taken to receive a response from physicians, health consumers may select to pay a fee to attract specialists on health Q&A websites, thereby receiving preference. For patients, questions that receive many answers from health experts indicate highly valuable information. Rafaeli et al [38] identified that economic motivators are associated with the participation of experts on Google Answer. Similarly, the length of a question is considered to influence the quality of the response: questions with fewer sentences receive a greater number of useful responses than those with many sentences [39].

Thus, we assumed that the length of the question and the value of reward affects the number of answers received in online health Q&A websites, leading to our third research question: what is the influence of the value of reward and the length of question on the number of responses received?

## Methods

### Data Collection

**Figure 1:**
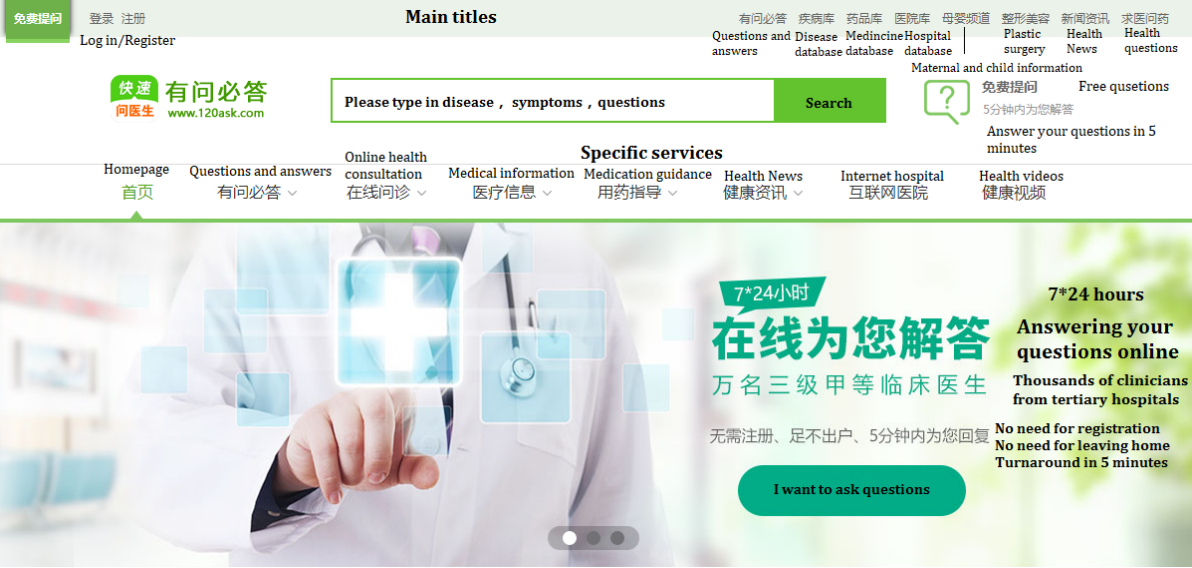
Homepage of the website “All questions will be answered” (accessed April 18, 2018).

Data were collected from a health Q&A website named “All questions will be answered” [15], one of China’s most popular online question and consultation websites. A screenshot of the homepage is shown in Figure 1. We selected this platform since it is widely adopted across mainland China and attracts a broad range of users. The platform was established in 2004, and over 3,000,000 physicians are currently using it to provide services to patients online. All physicians must be certified before they are able to respond to questioners, and more than 85% of them work in tertiary hospitals with rich clinical experience.

The health Q&A website is divided into various sections, separated by department and diseases, which facilitates access to specific questions and answers. Patients can obtain information on diseases, and can access patient tools and support for self-management [40]. Such a platform enabling uncomplicated access to health information is important in the management of chronic health conditions [31]. To simplify the analysis, in this study, we focused on patients with diabetes, which is considered one of the most common chronic diseases worldwide [41]. A large percentage of people with diabetes seek health information online [40]. These patients live with a common chronic disease and need to be able to self-manage and become an expert in their treatment [42]. We also selected to focus on hepatitis because many patients with hepatitis have chronic cases [43] and seek health information, especially surrounding treatment [44]. Therefore, questions were collected under the categories of diabetes and hepatitis, thereby excluding interference from other medical departments.

**Figure 2:**
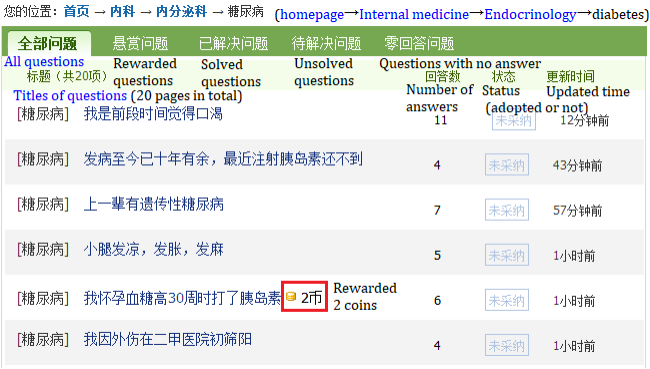
Listed questions under the category of diabetes (accessed March 23, 2018).

**Figure 3:**
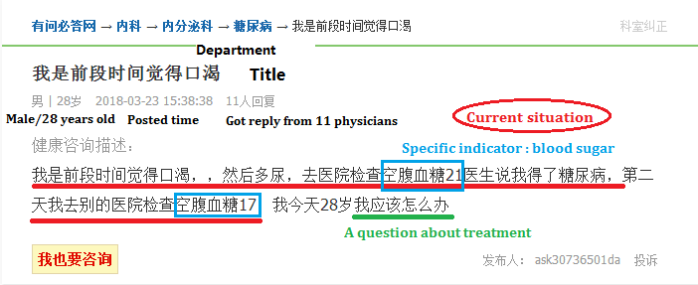
Example of a diabetes-related question (accessed March 23, 2018).

Users of the health Q&A website raise questions for themselves or for their friends and family members who are distressed by health problems. When they require an urgent reply, they may attract physicians by using “healthy coins,” which are exchanged with real money at a ratio of 1:1, as shown in Figure 2. Therefore, we classified the questions into free and rewarded questions. A predesigned Java-based Web crawler was used to obtain all questions under the category of diabetes and hepatitis in February 2018. The collected data are stored in a MySQL database. The website displays the most recent 4000 (200 pages multiplied by 20 questions per page) questions of each type. First, we obtained the URLs of questions under the two categories using page parsing and information extraction, and then collected the content of questions from these URLs; an example list of questions is shown in Figure 2 and the details of the first question in Figure 2 are shown in Figure 3. We acquired 4000 free questions and 4000 rewarded question under the topics of diabetes and hepatitis in January 2018.

After filtering for duplicate and invalid data by text preprocessing, the data comprised 3618 free and 3468 rewarded diabetes-related questions, and 3695 free and 2990 rewarded hepatitis-related questions. The collected data contained the content of each question, date the question was posted, number of answers received, and the age and gender of the patient who posted the question.

### Statistical Analysis

#### Overall Design

The data collected were divided into two parts. The first part was the content, including the text posted by patients suffering from chronic illnesses, which was explored through content analysis. The second part involved statistical data, including age of the patient, number of responses received to each question, and value of the reward. The gender of the patient was also included in the statistical part as a categorical variable. The length of the question was calculated by the number of words in the question, which was also included in the statistical part.

To answer research question 1, content analysis was performed to explore the text element of the question. A coding framework was developed with two main parts. The first consisted of a description of the condition; if the patient provided a description about their condition using specific biological indicators, we could consider that the patient is capable of describing the condition accurately. The other part was related to the phrasing of the content of the question. The content could be phrased as a question or a statement [36]. If one or more questions were included in the content, then we could infer that the patient is capable of phrasing the content as a question. The coding framework also included the classification of question type to address research question 2.

Linear regression analysis was conducted to address research question 3, and to explore the effects of the value of the reward and the length of question on the number of answers received to each question. SPSS 22.0 software (SPSS Inc., Chicago, IL, USA) was used to conduct these analyses.

#### Content Analysis

Oh et al [45] developed a coding schema for content analysis of health-related questions. The two types of information they considered were the information provided and the information sought. Participants provided a short description of their health conditions and problems and then asked question(s) about prevention, symptoms, diagnoses, or treatment of their diseases in most cases [45]. Based on this framework, and the conventional categories of questions, we developed a coding framework with two main parts: (1) description of the condition (provided information), and (2) whether or not the content was phrased as a question and the content of the question (information sought).

A sample of diabetes-related questions is provided in Figure 2. The text underlined in red is a description of a current situation, and the questions raised are underlined in green. The question providers commonly first describe the situation of the patient, and then raise the problems experienced, followed by a request for suggestions.

#### Coding Framework

**Figure 4:**
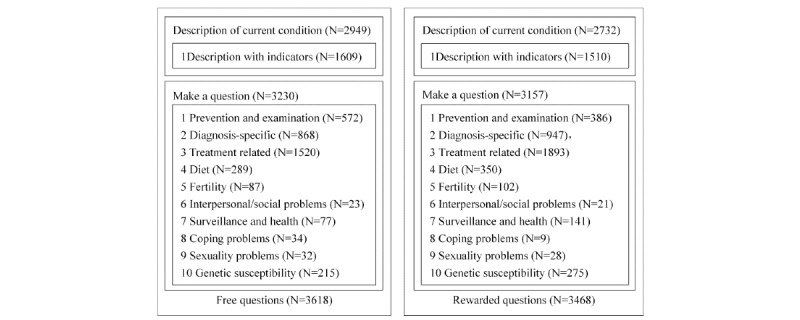
Coding results of diabetes.

The coding process was conducted by two research assistants following a training session. The two research assistants independently coded a random selection of 10% (362/3618, 347/3468; 370/3695, 300/2990) of the total number of questions within the pilot framework. If the result of the coding was different or they encountered concepts beyond the previous coding scheme, then the two research assistants discussed adjustment until a consensus was reached. After the independent coding was completed, the final coding framework was designed, and high intercoder reliability was demonstrated (Cohen kappa=0.80, 95% CI 0.55-1.05), indicating acceptable credibility. All questions were then coded using the newly developed framework; the coding framework is presented in Figure 4.

#### Descriptions

The descriptions mainly contained information relating to the patients’ current situation. A code of 1 was assigned when the question provider described the situation and a code of 0 was assigned when no description was provided. In the description group, we coded the description in the second layer. A code of 1 was assigned when the provider used specific indicators such as blood sugar and blood pressure in a question about diabetes or the value of viral DNA in a question about hepatitis.

#### Questions

Code 1 was assigned when the content included one or more question and code 0 was assigned for no question provided, and only a statement. The questions in group 1 were coded into 10 types after pilot coding. The classification referred to the studies of Oh [45], Rutten et al [34], and Fletcher et al [46], including: (1) prevention and examination, (2) diagnosis-specific, (3) treatment-related, (4) diet, (5) fertility, (6) interpersonal/social problem, (7) surveillance and health problem, (8) coping problem, (9) sexuality problems, and (10) genetic susceptibility problems. Code 1 was assigned when a specific content category was present, and code 0 was assigned for a question not related to these categories.

#### Linear Regression

The dependent variable was the number of responses received per question, which was estimated by the quantity of answers patients received from physicians after they had posted a question request on the health Q&A website. There were two independent variables: the first was the value of the reward, which was estimated by the number of “health coins,” and the second was the length of the question, which was calculated by the number of words. The patients’ gender and age were also included as control variables. To test our hypotheses against the value of reward and length of question, we formulated the following linear regression equation for the two diseases.

Answer*_i_* = α_0_ + α_1_Male*_i_* + α_2_Age*_i_* + α_3_Reward*_i_* + α_4_Length*_i_*

Where *i=*1,2…,n is the index of all patients raising questions, and α_0_ to α_4_ are the parameters to be estimated.

## Results

### Content Analysis

**Figure 5:**
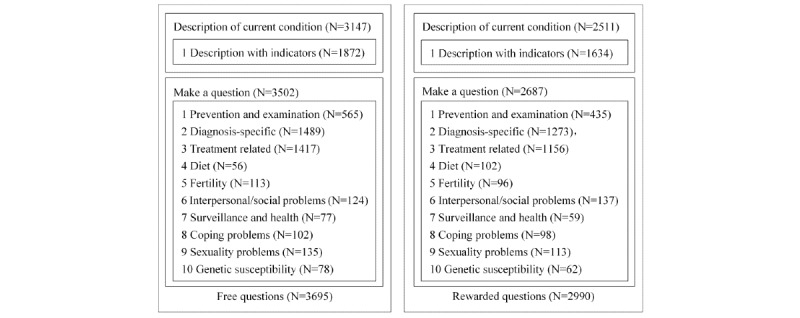
Coding results of hepatitis.

The coding results are shown in Figures 4 and 5. Most patients made a specific health request in terms of medical examination. Under the category of diabetes, the large majority of the free questions and reward questions contained a description of the patient’s condition. Under the category of hepatitis, the majority of free and rewarded questions included the condition of patients. Among the questions, just over half of diabetes or hepatitis question providers used one or more inspection indicators to describe the condition of patients, such as the blood sugar level. For example, in the questions shown in Figure 2, the man felt thirsty and experienced more frequent urination; he described his symptoms and reported his blood sugar level. Among all questions, 89.27% (3230/3618) and 94.77% (3502/3695) of free questions, and 85.17% (3147/3695) and 89.97% (2687/2990) of rewarded questions contained a specific request with interrogative sentences for the diabetes and hepatitis group, respectively. No specific information was provided in the remaining questions. For example, a patient raised the following question:

Recently, I found that I had a high blood sugar level after a medical examination. I like to eat sweet food and rice, I rarely exercise in my daily life, and I pay little attention to my diet. I am worried that I will have diabetes one day.

The man only talks about his condition with declarative sentences, and no specific question is posed. We might infer that he wanted to know whether or not he is likely to experience diabetes in his lifetime or how to prevent it, but he did not ask a question directly.

Information on prevention, examination, diagnosis, and treatment was most frequently requested by patients under both disease categories. In particular, the main concern for patients with diabetes was related to treatment, whereas patients with hepatitis more frequently asked questions focused on diagnosis. Patients commonly raised more than one question in their request. For example, one patient posted the following request:

My mother is 61 years old, and a medical examination indicated that her fasting blood glucose is 8.5. This indicator is now regularly tested using a home glucose meter, her fasting plasma glucose is about 9.8, and the number raises to 10 in two hours after a meal. Does my mother have diabetes and how do I treat it?

Patients with diabetes also paid attention to problems relating to diet, genetic susceptibility, fertility, surveillance, and health. Those with hepatitis cared more about sexuality, interpersonal/social issues, coping, and fertility problems.

### Differences Between Free and Rewarded Questions

Like many other expert-based Q&A websites, the “All questions will be answered” health Q&A website includes a pricing system. That is, patients may pay to receive answers to the questions they ask. We compared the information of questions that were answered freely with those that were rewarded using the Mann Whitney *U* test. Female patients were seen to be more likely to post questions in the health Q&A website than males. The length of questions was calculated by the number of words in each question [39]. The distributions of age and average length of question were significantly different in the two disease groups. Free questions attracted more answers than rewarded questions. Further details are provided in Table 1.

**Table 1:** Descriptive data and differences between free and rewarded questions.

Variable		Diabetes			Hepatitis	
		Free question	Rewarded question	Free question		Rewarded question
**Gender of patient**						
	Male	1673	1068	1514		996
	Female	1945	2400	2181		1994
	*P* value^a^	<.001			<.001	
**Age of patient (years)**						
	Min	0	0	0		0
	Max	98	89	92		91
	Mean	42.98	30.46	31.44		18.26
	SD	17.53	25.72	14.36		17.44
	*P* value^a^	<.001			<.001	
**Length of question (number of words)**						
	Min	12	4	10		4
	Max	745	1772	430		3527
	Mean	55.09	122.58	58.92		132.13
	SD	34.13	132.96	32.28		190.03
	*P* value	<.001			<.001	
**Number of responses**						
	Min	1	1	1		1
	Max	29	69	16		31
	Mean	4.73	2.97	4.55		3.33
	SD	2.08	3.14	1.47		2.72
	*P* value	<.001			<.001	
**Value of reward (“healthy coins”)**						
	Min	N/A^b^	2	N/A		2
	Max	N/A	310	N/A		200
	Mean	N/A	22.59	N/A		24.43

^a^*P* values are based on the Mann Whitney *U* test.

^b^N/A: not applicable.

### Linear Regression

Table 2 presents the results of the linear regression analysis based on ordinary least squares. Equations are presented in hierarchical order. First, the results are shown with only control variables in Model 1, and then the independent variables were added to Model 2. The adjusted *R^2^* and *F* values indicated a good overall fit. The coefficients for value of the reward were significant (*P*<.001) and negative, and the length of questions was significant for diabetes (*P*<.01) but not for hepatitis (*P*=.64).

**Table 2:** Linear regression for number of answers.

Independent variable	Diabetes (N=3468)		Hepatitis (N=2990)	
	Model 1 B (95%CI)	Model 2 B (95%CI)	Model 1 B (95%CI)	Model 2 B (95%CI)
Constant	2.663 (2.499-2.827), *t*=31.765, *P<*.001	3.288 (3.059-3.516), *t*=28.244, *P*<.001	2.356 (2.221-2.491), *t*=34.187, *P*<.001	3.541 (3.343–3.740), *t*=34.923, *P*<.001
Gender	0.333 (0.092-0.574), *t*=2.709, *P*=.007	0.296 (0.057-0.536), *t*=2.429, *P*=.02	0.705 (0.485-0.926), *t*=6.263, *P*<.001	0.604 (0.392-0.817), *t*=5.578, *P*<.001
Age	0.007 (0.002-0.011), *t*=3.074, *P*=.002	0.007 (0.002-0.011), *t*=3.096, *P*=.002	0.040 (0.034-0.046), *t*=13.293, *P*<.001	0.029, (0.02-30.034), *t*=9.469, *P*<.001
Value of reward	—^a^	–0.016 (–0.20 to –0.011), *t*=–6.572), *P<*.001	—	–0.038 (–0.056 to –0.031), *t*=–15.413, *P*<.001
Length of questions	—	–0.001 (–0.002-0.000), *t*=–2.764, *P*=.006	—	0.000 (–0.001–0.000), *t*=–0.475, *P*=.64
Adjusted *R*^2^	0.007	0.023	0.085	0.180
*F*	12.773	21.156	186.432	164.380

^a^Not applicable.

## Discussion

### Patients’ Abilities to Describe Their Illness and Phrase the Condition as a Question

Most of the patients were found to be capable of describing their personal condition. More than 40% of patients with diabetes and more than 50% of patients with hepatitis could describe their illness accurately by using indicators in their description. More than 85% of patients phrased their condition as a question overall.

Health literacy has received substantial attention [47,48] in the field of online health care. The ability to obtain, manage, and understand health information needs will help patients make more appropriate health choices in the future. To date, most health literacy studies have focused on the evaluation of health-related content or eHealth services [48], but the patient’s ability to seek appropriate information, especially when asking on a health Q&A website, is rarely investigated. Making an online request on a health Q&A website is different from a traditional offline face-to-face consultation. In a hospital setting, physicians can ask patients about their current condition and obtain crucial data for improved diagnosis and treatment. As this is a dynamic process, the physician can make adjustments during the interaction as needed. Most question providers followed this offline process and led with a description about their conditions. When they described the condition, they were also accustomed to using indicators, especially the blood sugar level as an important parameter for patients suffering from diabetes. In line with the old Chinese saying “prolonged illness made the patient a doctor,” most of the patients or their caregivers are able to consciously describe their condition and raise a question about the problems they are experiencing. Nevertheless, some users cannot phrase their condition as a question, such as the man who was afraid of getting diabetes, and these patients might require a platform to express their feelings. The less formal communication element of the health Q&A website may be more suitable for these types of patients. The platform should also provide guidance for users who cannot accurately express their question, and a questioning assistant tool might be helpful in this regard.

### Differences Between Patients With Diabetes and Hepatitis

Patients always seek information related to a specific illness or disease [29], and different diseases lead to various health concerns. Patients with diabetes mainly sought answers about treatment, followed by diagnosis, whereas those with hepatitis were more concerned about diagnosis, followed by treatment. Medical treatment information has always been searched most frequently [30]. The differences could be attributed to the characteristics of the two diseases: diabetes is a chronic disease, whereas some types of hepatitis are infectious. Patients with chronic diseases would be most interested in trying to find a cure or prevent disease progression, whereas those with infectious diseases may experience pressure on their social life. For the susceptible population, prevention and diagnosis are considered crucial.

For patients with diabetes, questions related to diet attracted the most attention, which is consistent with the study of Kuske et al [35]; this phenomenon might be attributed to the high sugar in foods and drinks. Patients with hepatitis also paid attention to diet but less so than patients with diabetes; their main concern with respect to diet was related to alcohol consumption, which is associated with the liver. Traditional Chinese diets are typically of ancient origin and are accepted by many Chinese people, which may explain the high number of questions about food and drink. Patients with diabetes were also concerned about other topics such as genetic susceptibility, fertility, and surveillance problems because genetic factors are the main cause of diabetes. Those with hepatitis also paid attention to sexuality, interpersonal, and fertility problems. Given that many types of hepatitis are infectious, the disease will affect the daily life and social interactions of patients. The present results enable health care website managers or physicians to understand patients’ information needs on health care. To improve the usefulness of health care provider websites, managers can divide the questions into minor groups and make the questions more targeted. Physicians can also be grouped into different types, allowing them to answer questions with which they are most familiar. Considering that treatment and diagnosis are frequently referred to, health care websites can also focus on these parts and offer educational articles about these topics.

### Factors Influencing Answer Provision

When patients or caregivers put forward their questions on a health Q&A website, they want informed answers to allow them to make the right decision about their health. Patients can attract answers from physicians in two ways. One is to textually describe more aspects about their situation, and the other is to pay a fee for the response. Our results indicate that the length of question negatively affected the number of responses received only in the diabetes group, and this finding is partly consistent with the study of Morris et al [39]. The fact that the length of questions had no significant effect on the number of responses received in the hepatitis group may be attributed to the characteristics of the data, as the maximum value of the number of questions was very large (3527); therefore, these outliers may have affected the results. In the diabetes group, the length of question affected the number of questions negatively; however, the adjusted *R^2^* value was quite low compared with that of the hepatitis group. More significant factors in the model may lead to a smaller adjusted *R^2^* value, indicating that further studies are needed to verify the effect of the length of question. The coefficients were also relatively low, which may be attributed to the large sample size.

There are two possible reasons for the finding that long sentences may lead to fewer responses. First, when patients face a complicated problem, they need to use many words to make their description clear, which might make it difficult for the physicians to understand or answer the questions. Second, too many questions may be included, which require additional time and effort from physicians. Raising a question with an appropriate length of sentence is difficult. For health Q&A website users who want to get answers quickly, they should focus on refining their content and make their questions more specific. Providing a training program for patients may also be helpful for users to raise a proper, understandable question.

Some question providers pay a fee when they raise a question; however, the free questions generally received more answers than those that are rewarded. This result is in contrast to the finding of Rafaeli et al [38], who investigated economic and social motivators; hence, we must include additional factors in our further research. The amount of reward is negatively related to the number of answers. People who offer a reward are likely to make the question longer, thereby receiving fewer answers. The pricing mechanism is not clear enough for users of the website. They change their money into “health coins” at a ratio of 1:1, but whether or not these “health coins” are fairly given to the specific physicians is unknown. Therefore, the health Q&A website should provide more suitable motivation mechanisms to encourage physicians to participate on the website more frequently.

### Limitations

This study has some limitations. First, the questions were only collected from one website, leading to limitations in the data source setting. The data collected might only reflect the needs of specific online users, and the conclusions may not apply to patients with other diseases or those that use other health care platforms. Future research should collect data from other platforms and make comparisons among different data sources. Second, because the proportion of free questions was higher than that of rewarded questions, the time periods were different in the two groups. Hence, the time factor may also have affected the results. Third, we may have missed some potential question types in our developed framework as some words may have more than one meaning in Chinese. Finally, the effect of descriptive variables on the willingness of physicians to answer questions should be further considered.

### Conclusion

Health literacy requires many abilities in relation to health information and decision making. Our study provides insight into the ability of patients to make a suitable question request on expert health Q&A websites. The examination of patients’ health topics helps to provide a better understanding of the information needs relating to different diseases, and different health information should be made available to targeted patients based on their needs. A negative effect of the length of question and value of the reward on the number of responses received was identified. Patients do not need to pay a fee to receive a response to their questions and should aim to refine the length of their questions if they want more timely responses from specialists.

## Abbreviations

Q&A: question and answer
